# Macroscopic Evaluation of Gastric Specimens After Laparoscopic Sleeve Gastrectomy—an Optimum Screening Test for Incidental Pathologies?

**DOI:** 10.1007/s11695-018-3485-4

**Published:** 2018-09-05

**Authors:** Maciej Walędziak, Anna Różańska-Walędziak, Michał R. Janik, Krzysztof W. Paśnik, Piotr K. Kowalewski

**Affiliations:** 1 Department of General, Oncological, Metabolic and Thoracic Surgery, Military Institute of Medicine, Szaserów 128 St., Warsaw, 04-141 Poland; 2 2nd Department of Obstetrics and Gynecology, Medical University of Warsaw, Warsaw, Poland

**Keywords:** Obesity, Bariatric surgery, Laparoscopic sleeve gastrectomy, Histopathology, Pathological findings

## Abstract

**Introduction:**

Obesity is a serious lifestyle disease with various comorbidities and an augmented risk of cancer. Laparoscopic sleeve gastrectomy (LSG) has recently become the most popular bariatric procedure worldwide. While the cost-effectiveness is a major healthcare providers’ concern, the point of histological exam of each resected tissue may be questioned.

**Material/Methods:**

We prospectively included patients who underwent LSG. Before the surgery, gastroscopy and abdominal sonography were performed to exclude malignancies. The gastric specimen was cut open after the surgery and inspected macroscopically, then sent for a microscopic examination.

**Results:**

In 5 cases out of 115, macroscopic evaluation of the resected specimen performed by the surgeon suggested existing pathology, confirmed by a microscopic evaluation in 3 out of 5 cases. In the remaining 2 cases, pathological analysis did not reveal abnormalities. In 110 cases, the gastric specimen was recognized to be unchanged by the surgeon, 109 out of which were confirmed by the pathologist to be normal, in 1 case a hyperplastic polyp was found. The sensitivity of macroscopic evaluation reached 75% (95% CI, 19.4–99.4%, *p* = 0.625), with specificity of 98.2% (95% CI, 93.6–99.8%, *p* < 0.0001), and negative predictive value of 99.1% (95% CI, 95–99.9%, *p* < 0.0001).

**Conclusions:**

During LSG, a thorough visual inspection of the peritoneal cavity along with a macroscopic surgical evaluation of specimen in patients who had preoperative endoscopy with no findings allows to achieve very good specificity and good sensitivity. Therefore, this procedure may be useful as a screening test for incidental pathologies in bariatric patients and may exclude unnecessary histological examination.

## Introduction

Obesity is a serious lifestyle disease with various comorbidities and a higher than average risk of cancer [1–4]. As it is commonly known, bariatric surgery is the most effective way of treatment [5] and the annual number of bariatric procedures performed constantly increases, having reached 600,000 worldwide in 2014 [6]. This global trend is also observed on our national level, as reported by Janik et al. [7].

Bariatric techniques are constantly evolving with new concepts emerging. Laparoscopic sleeve gastrectomy (LSG) has recently become the most popular procedure worldwide. As financial efficiency has become one of the healthcare providers’ major concerns, the point of histological exam of each resected tissue specimen can be questioned. The utility of histological examination of the resected macroscopically unchanged appendix, gallbladder or hemorrhoids is doubted by some of the authors [8, 9]. Therefore, we wanted to analyze whether bariatric specimens should be examined, also considering the recent publications about incidental findings after LSG [10, 11].

Neither national, nor international bariatric recommendations present the optimum strategy of proceeding with the gastric specimen resected during laparoscopic sleeve gastrectomy.

Our aim was to evaluate whether manual macroscopic inspection of the gastric specimens is a useful tool to reveal incidental pathologies after LSG.

## Material and Methods

We prospectively included all consecutive patients who had undergone LSG from January to August 2017 in our bariatric center. The patients were qualified for bariatric surgery according to the acknowledged international criteria [12]. A gastroscopy and abdominal sonography were routinely performed before the surgery, and patients with visible malignancies were excluded and qualified for detailed evaluation. Every procedure was performed by a bariatric surgeon according to the protocol described in our previous studies [13].

Peritoneal cavity was inspected after insulation. In the case of any suspicious findings in the peritoneal cavity, a biopsy was performed for further evaluation. After the surgery, each gastric specimen was cut open and macroscopically inspected by the surgeon. The evaluation included visual analysis and palpation, and the result was presented as suspected pathology or normal tissue. Then each specimen was sent for a microscopic examination performed by two pathologists. The microscopic examination was performed after chemical fixation and processing with an addition of immunohistochemistry staining if necessary. The medical diagnosis was formulated as a pathology report by two independent pathology specialists.

We gathered the data on patients’ age, BMI, and weight loss before the surgery as well as the results of the macro- and microscopic evaluations of the specimen.

Statistical analysis was performed using SAS software, University Edition (SAS Institute Inc., Cary, NC, USA). To compare continuous variables, the Mann-Whitney *U* test and Student’s *t* test were used when appropriate. Statistical significance was set at *p* < 0.05. The study was approved by the Bioethical Committee of the Warsaw Military Institute.

## Results

Among 115 patients, 77 were female, the median age was 38 years (Q1, 31; Q3, 48). The mean BMI was 48.4 kg/m^2^ (± 8.4), and mean weight loss during the qualification process reached 10.6 kg (± 5). The mean length of hospital stay after the surgery was 2 days (± 0.3). Sixteen patients (13.91%) had previous history of peptic ulcers. Thirty-five (30.43%) patients admitted smoking (Table 1).

**Table 1:** Demographic data before surgery

	Value	% (SD) (range)
Gender (female/male)	71/44	61.7%/38.3%
Median age	38	(31–48)
Mean BMI (kg/m^2^)	48.4	(± 8.4)
Mean weight loss (kg)	10.6	(± 5)

In five cases, macroscopic evaluation of the resected gastric specimen performed by the surgeon suggested existing pathology, which was confirmed by a microscopic evaluation by a pathologist in three out of five cases (two cases of hyperplastic polyps and one case of neuroendocrine microtumor). In the remaining two cases, pathological analysis did not reveal abnormalities in the gastric wall. In 110 cases, the gastric specimen was recognized to be unchanged by the surgeon, 109 out of which were confirmed by the pathologist to be normal, in one case the pathologist found a hyperplastic polyp. Summarizing, the results of the microscopic analysis were negative in 111 cases and positive in four cases (Table 2).

**Table 2:** Macroscopic and microscopic evaluation

	Positive results	Negative results
Macroscopic evaluation (surgeon)	5	110
Microscopic evaluation (pathologist)	4	111

The sensitivity of the macroscopic surgical inspection reached 75% (95% CI, 19.4–99.4%, *p* = 0.625), with specificity of 98.2% (95% CI, 93.6–99.8%, *p* < 0.0001), and negative predictive value of 99.1% (95% CI, 95–99.9%, *p* < 0.0001) (Table 3).

**Table 3:** Contingency table

		Microscopic evaluation (pathologist)		
		Pathology	Clear	TOTAL
Macroscopic evaluation (surgeon)	Pathology	TP=3 (75%)	FP=2 (1.8%)	5
	Clear	FN=1 (25%)	TN=109 (98.2%)	110
	TOTAL	4	111	115

TP – true positive, FP – false positive, FN – false negative, TN – true negative

The microscopic evaluation revealed gastritis in 50 specimens (43.48%), 61 (53.04%) had normal mucosa, and 4 cases presented pathologies: 3 cases of hyperplastic polyps (2.61%) and 1 neuroendocrine microtumor (0.09%) with two G1 neuroendocrine tumor (NET) foci, with proliferation index lower than 1%, mitotic index lower than 1 and high chromogranine and synaptophysin reaction, and diffused neuroendocrine hyperplasia with dysplasia in the rest of the mucosa.

## Discussion

Bariatric surgery is a commonly accepted method of treatment of obesity when noninvasive methods are ineffective. Laparoscopic sleeve resection has recently become the most popular bariatric procedure worldwide.

Excess weight is proved to be a risk factor for malignancies and the incidence of gastrointestinal stromal tumors (GIST) is much higher in the obese population [14]. When not diagnosed in time, GISTs can lead to poor outcomes as 40% up to 50% of patients may have relapse even after oncologically radical resection [15]. Healthcare professionals are still debating whether or not a surgical specimen should be evaluated microscopically if it does not come from an oncological procedure. In the case of laparoscopic sleeve gastrectomy, the researchers present opposed approaches.

Hansen et al. [16] evaluated 351 tissue samples, not having revealed any pathologies, therefore concluding that a standard pathological examination is unnecessary. Having analyzed 546 microscopic results of gastric specimens, AbdullGaffar et al. [17] found four pathological cases (0.8%): two hyperplastic polyps, one cyst, and one benign tumor. Further investigation did not show any anomalies in patients, and the fact of positive findings did not increase the perioperative risk. Due to cost-effectiveness, the author suggested restraining from routine histopathological analysis of the tissue resected. Both authors agree that every resected gastric specimen should be evaluated by the surgeon macroscopically and by palpation, followed by a microscopic analysis in the case of positive findings.

On the contrary, Yardimci et al. [18] evaluated 755 specimens and found neoplasms in 4 (0.5%) cases. Canil et al. [19] analyzed 925 cases in a 5-year span with a 0.3% rate of neoplasms. Both authors state that both preoperative gastroscopy and postoperative pathological examination are crucial in the case of LSG. Similar conclusions are presented by Almazeedi et al. [20]. After having analyzed the histopathological results of 656 patients, he found 12 cases with abnormalities (1.8%). None of the researchers mentioned the sensitivity or specificity of the presented methods of evaluation of the resected gastric specimen after LSG.

Over the last few years, our team of researchers reviewed 1252 bariatric procedures performed over the course of 3 years [10, 11]. After laparoscopic visual evaluation during LSG, 50 specimens were qualified to further microscopic evaluation, revealing pathologies in 29 cases, with GISTs found in 16 cases. That may lead to the conclusion that GIST predominance is 1000 times higher among bariatric patients than in general population. We continued our research trying to create an optimal screening test, which would allow to select specimens for further evaluation. Introduction of such screening would reduce the cost of unnecessary routine histopathological exams. Our conclusions are similar to those presented by Gagner [21], who recommends surgical macroscopic evaluation of the specimens as standard after LSG. Every suspicion should be marked, described, and verified by the pathologist.

Any diagnostic test should have an acceptable sensitivity and specificity. Our method of macroscopic evaluation compared with microscopic histopathological examination revealed pathologies in 75% of cases and correctly identified unchanged specimen in 98.2% of cases. Our method also presented a high negative predictive value of 99.1%, which makes it usable as a screening test. The limitation of the study is a relatively low number of cases included so further studies with larger groups are necessary to evaluate the usefulness of the presented method.

## Conclusion

During LSG, a thorough visual inspection of the peritoneal cavity along with a macroscopic surgical evaluation of the gastric specimen in patients who had preoperative endoscopy with no macroscopic findings allows to achieve very good specificity and good sensitivity. Therefore, this procedure may be useful as a screening test for incidental pathologies in bariatric patients and may exclude unnecessary histopathological examination of all surgical specimens after bariatric operations.
